# Non-contact diagnosis of obstructive sleep apnea using impulse-radio ultra-wideband radar

**DOI:** 10.1038/s41598-020-62061-4

**Published:** 2020-03-24

**Authors:** Sun Kang, Dong-Kyu Kim, Yonggu Lee, Young-Hyo Lim, Hyun-Kyung Park, Sung Ho Cho, Seok Hyun Cho

**Affiliations:** 1 Department of Electronics and Computer Engineering, Hanyang University, Seoul, Republic of Korea; 2 Department of Otorhinolaryngology-Head and Neck Surgery and Institute of New Frontier Research, Chuncheon Sacred Heart Hospital, Hallym University College of Medicine, Chuncheon, Republic of Korea; 3 Division of Cardiology, Department of Internal medicine, College of Medicine, Hanyang University, Seoul, Republic of Korea; 4 Department of Pediatrics, College of Medicine, Hanyang University, Seoul, Republic of Korea; 5 Department of Otorhinolaryngology-Head and Neck Surgery, College of Medicine, Hanyang University, Seoul, Republic of Korea

**Keywords:** Sleep disorders, Laboratory techniques and procedures

## Abstract

While full-night polysomnography is the gold standard for the diagnosis of obstructive sleep apnea, its limitations include a high cost and first-night effects. This study developed an algorithm for the detection of respiratory events based on impulse-radio ultra-wideband radar and verified its feasibility for the diagnosis of obstructive sleep apnea. A total of 94 subjects were enrolled in this study (23 controls and 24, 14, and 33 with mild, moderate, and severe obstructive sleep apnea, respectively). Abnormal breathing detected by impulse-radio ultra-wideband radar was defined as a drop in the peak radar signal by ≥30% from that in the pre-event baseline. We compared the abnormal breathing index obtained from impulse-radio ultra-wideband radar and apnea–hypopnea index (AHI) measured from polysomnography. There was an excellent agreement between the Abnormal Breathing Index and AHI (intraclass correlation coefficient = 0.927). The overall agreements of the impulse-radio ultra-wideband radar were 0.93 for Model 1 (AHI ≥ 5), 0.91 for Model 2 (AHI ≥ 15), and 1 for Model 3 (AHI ≥ 30). Impulse-radio ultra-wideband radar accurately detected respiratory events (apneas and hypopneas) during sleep without subject contact. Therefore, impulse-radio ultra-wideband radar may be used as a screening tool for obstructive sleep apnea.

## Introduction

Obstructive sleep apnea (OSA) is characterized by repetitive episodes of apnea and/or hypopnea and various degrees of hypoxia caused by upper airway collapse during sleep. Many studied showed that the OSA is associated with several cardiovascular complications^1,2^. OSA may also be associated with depression, neuropsychological effects and structural changes in the brain^3,4^. The gold standard examination for the diagnosis of OSA is attended, in-laboratory, full-night polysomnography (PSG) with multichannel monitoring^5^. However, PSG requires many attachments to analyse the patterns of sleep in each patients. Thus, some patients have difficulty with achieving satisfactory sleep due to these obstructive circumstances. Moreover, analysis of PSG data must consider the first-night effect caused by unfamiliar sleep circumstances and discomfort due to restricted movement resulting from the numerous leads placed on the patient^6,7^.

To overcome these problems, several non-contact devices have been developed for the estimation of the obstructive apnea–hypopnea Index (AHI)^8–12^. In addition to these tools, impulse radio ultra-wideband (IR-UWB) radar sensors have recently been proposed as a potentially viable tool to monitor and measure body movements. By transmitting and receiving an impulse signal which occupies wide bandwidth in frequency domain, IR-UWB radar can recognize targets without contact. Because the IR-UWB radar uses wide bandwidth and high carrier frequency, it has several advantages such as high resolution, good penetration, small antenna size, and simple hardware structure. Thus, without touching the body, IR-UWB radar sensors can detect not only large movements of the human body but also small movements such as breathing. Moreover, the IR-UWB radar emits impulse signals with very low power, which is not harmful to the human body. Due to these characteristics, several studies have described various clinical applications using IR-UWB radar^13–17^.

Therefore, in the present study, we developed a novel sleep assessment tool using an IR-UWB radar sensor. To validate its agreement and accuracy, we compared the scoring of routine American Academy of Sleep Medicine-compliant PSG data to that of data obtained from the IR-UWB radar sensor within the same OSA patients.

## Methods

### Subjects

We enrolled 99 subjects with symptoms consistent with suspected OSA (e.g., excessive daytime sleepiness, loud snoring, or observed apnea episodes) who were referred to the sleep laboratory at Hanyang University Hospital between November 2017 and October 2018. Among those, five patients were excluded due to short sleep time. Finally, a total of 94 eligible subjects were included in this study. We used the Epworth Sleepiness Scale and the Pittsburgh Sleep Quality Index to assess daytime sleepiness and sleep quality, respectively. This study was approved and the study processes were monitored by the Institutional Review Board of Hanyang University Hospital (No. 2017-05-004-001). Written informed consent was obtained from all enrolled subjects. All methods were performed in accordance with relevant guidelines and regulations.

### Polysomnography

An overnight PSG (Alice 5; Philips Respironics, Amsterdam, Netherlands) was performed to record the respiratory events and determine the presence of OSA. According to recent criteria in the American Academy of Sleep Medicine manual, the apnea–hypopnea index (AHI) was manually scored by a trained sleep technician^5^. Apnea in adults was defined as a drop in the peak signal excursion of ≥90% from that of pre-event baseline using an oronasal thermal sensor for ≥10 seconds. Hypopnea in adults was defined as a peak signal excursion drop of ≥30% from that of pre-event baseline using nasal pressure for ≥10 seconds in association with either ≥3% arterial oxygen desaturation or an arousal. OSA was diagnosed with an AHI ≥ 5 and OSA severity was classified into three groups; mild OSA (5 ≤ AHI < 15), moderate OSA (15 ≤ AHI < 30), and severe OSA (AHI ≥ 30).

### Non-contact respiratory monitoring using IR-UWB radar

**Figure 1:**
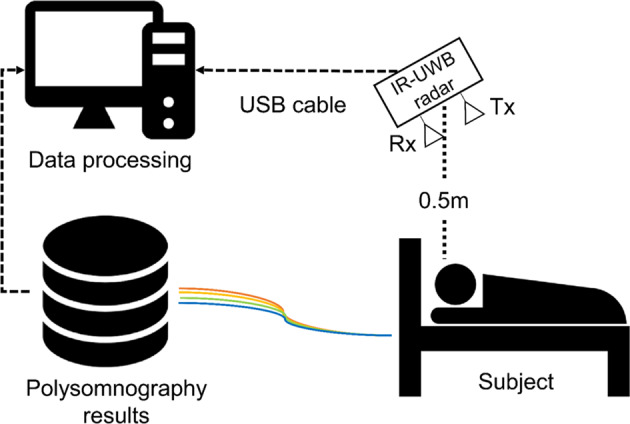
Illustration of how to obtain sleep data from IR-UWB radar sensors and polysomnography.

**Figure 2:**
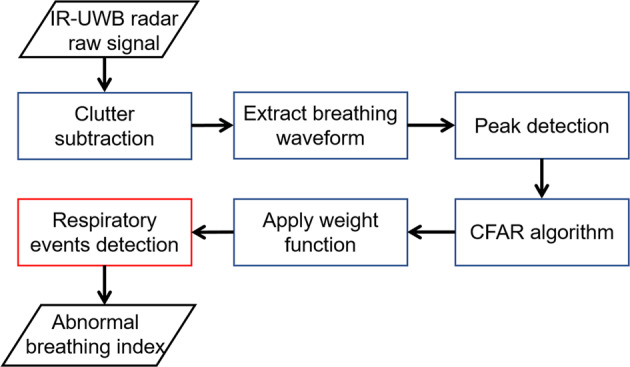
Algorithm for the detection of respiratory events from IR-UWB radar.

The IR-UWB radar sensor (XK300-SA; Xandar Kardian, Delaware, USA) was placed 0.5 m from the patient’s head (Fig. 1) and raw data were simultaneously obtained from radar during full-night PSG. The radar signals were processed by MATLAB (Mathworks, Natick, USA) and we developed an algorithm to detect abnormal respiratory events, as shown in Fig. 2. Because the raw data were biased by various external signals, so-called clutter (e.g., echo signals from the walls, ceilings, and other obstacles) was removed using a background subtraction algorithm^18^. We then extracted breathing waveforms based on our previous experiment^19^. The peak points (upper and lower) were also extracted from the breathing waveforms to discriminate apnea and hypopnea events from normal breathing. Next, a constant false alarm rate (CFAR) algorithm was used to set the baseline of the breathing waveform^20^. In this study, the respiratory event (abnormal breathing) was defined as a drop in the peak in the radar signal by ≥30% from that of the pre-event baseline which lasted for longer than 10 seconds.

### Pilot test of the CFAR algorithm with or without weight function adaptation

**Figure 3:**
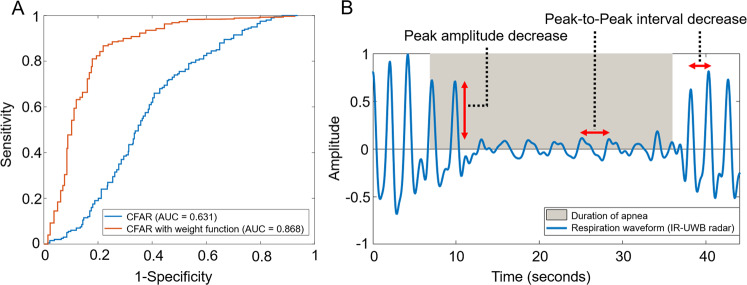
The effect of weight functions on the performance of the constant false alarm rate (CFAR) algorithm: (**A**) Receiver operating characteristic (ROC) curve for confirming the performance of the CFAR algorithm. (**B**) A decreased peak amplitude and peak-to-peak interval of the breathing waveform was observed during the apneic period by IR-UWB radar. After applying the weight function, the ROC curve of the CFAR algorithm increased.

To confirm the performance of the proposed CFAR algorithm, a pilot test was performed on 770 normal breathing patterns and 287 abnormal breathing events including apnea and hypopnea. A receiver operating characteristic (ROC) curve of the CFAR algorithm was drawn (Fig. 3A). The area under the ROC curve was as low as 0.631; therefore, we further analysed the patterns of the respiratory events to improve the performance. We found that radar-detected abnormal breathing included variabilities in terms of amplitude and frequency. The interval of abnormal breathing was preceded by a variable decrease in radar amplitude followed by a variable increase in radar frequency (Fig. 3B). After applying these weight functions, the performance of IR-UWB radar (area under the ROC curve) to detect respiratory events increased to 0.868 (Fig. 3A). We calculated the abnormal breathing index (ABI), defined as the number of radar-detected respiratory events per hour of sleep, to compare with AHI.

### Statistical analysis

Continuous variables were expressed as means ± standard deviation (SD) and categorical variables were expressed as numbers (%). Kruskal–Wallis tests were used employed to check for statistically significant differences in the baseline characteristics of subjects. Intraclass correlation coefficients R and Bland–Altman plots with 2.5% and 97.5% limits of agreement (LOA) were used to assess the agreements between the ABI from radar and the AHI from PSG. Single-sample t-tests were performed to evaluate the bias between the ABI and the AHI. To check the diagnosis accuracy of OSA severity, a confusion matrix for control, mild, moderate, and severe OSA was applied. All statistical analyses were conducted using the statistical software in R-3.5.1 (R Foundation for Statistical Computing, Vienna, Austria). *P*-values <0.05 were considered statistically significant.

## Results

### Subject characteristics

Of the 94 enrolled subjects, 71 (male, 62; female, 9) were diagnosed with OSA and 23 (male, 14; female, 9) were diagnosed as non-OSA. The OSA patients consisted of 24, 14, and 33 subjects with mild, moderate, and severe OSA, respectively. The baseline clinical and sleep parameters for enrolled are presented in Table 1.

**Table 1:** Demographic data and sleep parameters of the enrolled population.

	Control (N = 23)	Mild OSA (N = 24)	Moderate OSA (N = 14)	Severe OSA (N = 33)
**Subject characteristics**				
*Age (years)*	32.2 ± 16.8	45.4 ± 12.7^*^	43.3 ± 10.6	50.2 ± 12.4^*^
*Body mass index (kg/m*^2^)	23.4 ± 3.6	25.5 ± 2.3^*^	27.1 ± 2.3^*^	27.3 ± 2.9^*^
*Neck circumference (cm)*	35.2 ± 3.4	38.9 ± 3.8^*^	39.7 ± 2.6^*^	39.6 ± 3.7^*^
*ESS*	8.1 ± 5.4	7.8 ± 5.1	6.3 ± 5.6	8.8 ± 5.2
*PSQI*	7.25 ± 5.1	8.6 ± 4.5	6.7 ± 3.4	7.9 + 3.8
**Polysomnographic data**				
*Total sleep time (min)*	331.1 ± 51.6	326.9 ± 50.4	318.9 ± 35.9	286.8 ± 59.9^*†^
*Sleep efficiency (%)*	83.2 ± 18.9	85.8 ± 10.7	81.9 ± 8.5	77.9 ± 11.3^*†^
*N3 (%)*	11.2 ± 11.3	2.1 ± 5.4^*^	0.8 ± 1.3^*^	0.9 ± 2.5^*^
*REM (%)*	16.4 ± 6.3	17.3 ± 6	18.7 ± 4.4	14.3 ± 6.3^‡^
*AI (events/hr)*	0.4 ± 0.7	2.9 ± 2^*^	6 ± 4.6^*^	16.4 ± 6.3^*†‡^
*HI (events/hr)*	1.4 ± 1.1	2.9 ± 6.3^*^	14.1 ± 5.5^*†^	30.9 ± 20.9^*†^
*AHI (events/hr)*	1.9 ± 1.4	9.5 ± 2.6^*^	20.1 ± 4.2^*†^	53.9 ± 16.9^*†‡^
*RERA index (events/hr)*	1.9 ± 2.2	4.1 ± 3.4^*^	5.9 ± 4.6^*^	2.4 ± 2.6^†‡^
*RDI (events/hr)*	3.7 ± 3.2	13.6 ± 4.1^*^	26.1 ± 7.3^*†^	56.3 ± 16.3^*†‡^
*Arousal index (events/hr)*	24.2 ± 12.5	33.6 ± 13.3^*^	42.5 ± 11.7^*^	67.9 ± 23.8^*†‡^
*Lowest O*_2_ *(%)*	91.8 ± 2.1	82.3 ± 8.2^*^	81.9 ± 8.3^*^	74.4 ± 9.1^*†‡^
*Mean O*_2_ *(%)*	96.7 ± 0.8	95.7 ± 1.3^*^	95.1 ± 1.7^*^	94.1 ± 1.9^*†^

OSA, obstructive sleep apnea; ESS, Epworth Sleepiness Scale; PSQI, Pittsburgh Sleep Quality Index; AI, Apnea Index; HI, Hypopnea Index; AHI, Apnea–Hypopnea Index; RERA, Respiratory Effort-related Arousal; RDI, Respiratory Disturbance Index.

*p < 0.05 vs. control, ^†^p < 0.05 vs. patients with mild OSA, ^‡^p < 0.05 vs. patients with moderate OSA.

### Detection of respiratory events by IR-UWB radar

**Figure 4:**
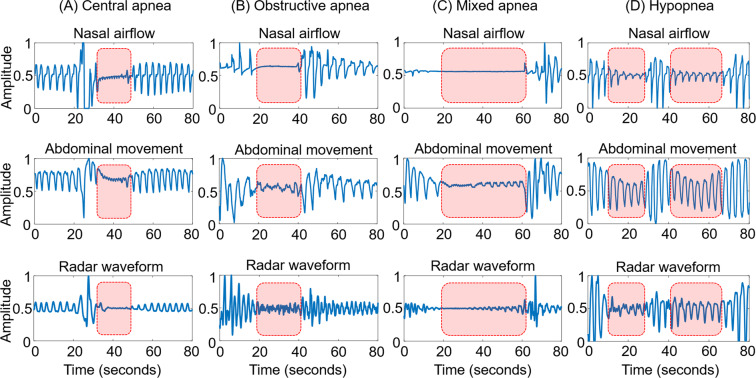
Normalized respiratory signals from PSG (nasal airflow and abdominal movement) and IR-UWB radar: IR-UWB radar can be used to detect three types of apneas (**A**) central, (**B**) obstructive, (**C**) mixed, and (**D**) hypopnea.

Three types of apnoea (central, obstructive, and mixed) and hypopnea could be detected by IR-UWB radar (Fig. 4). To compare all measurements (nasal airflow, abdominal movement, and radar waveform), we normalized the amplitude of the respiration waveforms (from 0 to 1). There was a total loss of radar waveform in central apnea (Fig. 4A) and a marked decrease in the radar waveform in obstructed apnea (Fig. 4B). Mixed apnea showed an initial central followed by obstructive apneas (Fig. 4C). Hypopnea was also identified by IR-UWB radar (Fig. 4D). In this study, all apneas and hypopneas were recorded as abnormal breathing.

### Agreement between ABI and AHI

**Figure 5:**
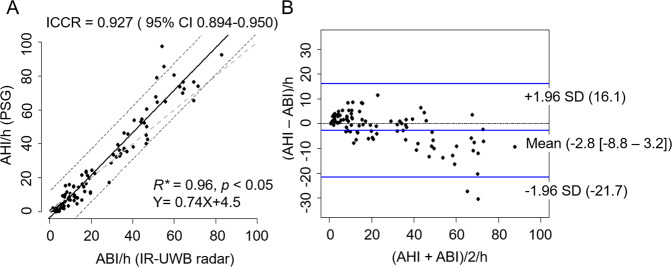
Comparisons of apnea-hypopnea index (AHI obtained from PSG) and abnormal breathing index (ABI obtained from the IR-UWB radar). (**A**) Scatter plots of ABI versus AHI. (**B**) Bland-Altman plots for visualization of the agreement between AHI and ABI. Lines indicate the average difference and the 2 standard deviations (Intraclass correlation coefficients R, ICCR; Confidence interval, CI; Standard deviation, SD).

To evaluate the agreement between ABI from IR-UWB radar and AHI from PSG, we used correlation (Fig. 5A) and Bland–Altman (Fig. 5B) plots. Excellent agreement was found between the ABI and AHI (Intraclass correlation coefficients R = 0.927, 95% confidence interval [0.894–0.950]; Fig. 5A). Moreover, the Bland–Altman plots showed low mean biases (−2.8 [−8.8–3.2]) and good LOA (lower LOA = −21.7, upper LOA = 16.1; Fig. 5B). Subgroup analysis according to OSA severity revealed that the mean value of the absolute difference between AHI and ABI was smallest in mild OSA (mean = −2.03), whereas the largest mean value of those was found in severe OSA patients (mean = 9.46) (Supplementary Table 1). The LOA width was shortest in normal subjects (lower LOA = −5.58, upper LOA = 0.99), whereas patients with severe OSA showed the longest LOA width (lower LOA = −14.53, upper LOA = 33.46).

### Diagnostic accuracy according to OSA severity

**Figure 6:**
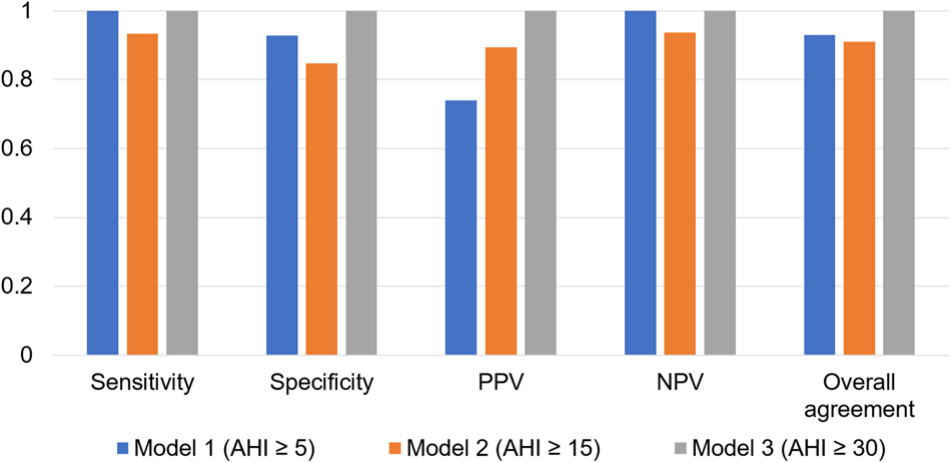
Performance of three models of IR-UWB radar for the diagnosis of obstructive sleep apnea. Sensitivity, specificity, positive predictive value (PPV), negative predictive value (NPV), and overall agreement were assessed.

Using the binary classifiers at AHI of 5, 15, and 30, the positive predictive value, negative predictive value, sensitivity, specificity, and overall agreement were examined (Fig. 6). Models 1, 2, and 3 were defined based on AHI cut-off values of 5, 15, and 30, respectively. The positive predictive value was 74% in Model 1, 89% in Model 2, and 100% in Model 3, whereas we observed the negative predictive value were 100%, 94%, and 100%, respectively. The sensitivity of the ABI from IR-UWB radar was 100% in Model 1, 93% in Model 2, and 100% in Model 3, whereas the specificities were 92%, 84%, and 100%, respectively. The overall agreement for AHIs of 5, 15, and 30 were 0.93, 0.91, and 1, respectively.

When we performed OSA severity based on ABI threshold defined as mild OSA (5 ≤ ABI < 15), moderate OSA (15 ≤ ABI < 30), and severe OSA (ABI ≥ 30), 85% subjects were matched to the OSA severity based on AHI score (Table 2). The precision value was 0.74 in control group, 0.79 in mild OSA group, 0.78 in moderate OSA group, 1 in severe OSA group, whereas the recall value was 1 in control group, 0.68 in mild OSA group, 0.69 in moderate OSA group, 1 in severe OSA group. The most common misdiagnosis of the IR-UWB radar was that of mild OSA diagnosed in normal subjects (N = 6). Interestingly, there was no misdiagnosis by IR-UWB radar of patients with severe OSA.

**Table 2:** Data accuracy between polysomnography (PSG) and impulse-radio ultra-wideband (IR-UWB) radar according to the severity of obstructive sleep apnea (OSA).

PSG (AHI)	IR-UWB radar (ABI)				Recall
	Control	Mild OSA	Moderate OSA	Severe OSA	
Control	17	0	0	0	1
Mild OSA	6	19	3	0	0.68
Moderate OSA	0	5	11	0	0.69
Severe OSA	0	0	0	33	1
Precision	0.74	0.79	0.78	1	**0.85**

The severity of OSA was categorized as follows: control (ABI or AHI < 5), mild (5 ≤ ABI or AHI < 15), moderate (15 ≤ ABI or AHI < 30), and severe (ABI or AHI ≥ 30).

ABI, Abnormal Breathing Index; AHI, Apnea–hypopnea Index.

The value of the overall agreement is 0.85.

## Discussion

Previously, we have investigated the usefulness of IR-UWB radar in various areas of industry and in medical fields^14,15,17^. IR-UWB radar is a safe and non-contact sensor that allows the monitoring of subject vital signs; therefore, we applied this technology in the present sleep study. Interestingly, we found an excellent agreement between ABI (IR-UWB radar) and AHI (PSG) and that IR-UWB radar could diagnose the presence of OSA regardless of its severity. Therefore, IR-UWB radar could be used as a screening tool for sleep apnea.

During OSA diagnosis, PSG is usually performed with multichannel monitoring; thus, it requires continuous signal adjustment by sleep technicians for optimal recording. For this reason, patients often experience an inconvenient environment rather than home sleep and often have difficulties in falling into a deep sleep. Several investigators have sought to develop non-contact devices to replace PSG, such as acoustic analysis, pressure sensors, and infrared thermography^8–12^. Among those, a snoring sound sensor using a microphone is simple for patients to operate without body contact. While such microphones are more convenient, body posture during sleep may affect the acoustic characteristics of snoring and the protection of the subject’s privacy is difficult due to the microphone. Additionally, the pressure sensor can detect the breathing signal due to mass movement. Thus, it may actually be more indicative of respiratory effort. However, the sleep assessment tool based on pressure sensor has critical limitations. It requires multiple sensors for accurate measurement and subjects must sleep in specific locations such as a bed attached several pressure sensors. Moreover, a higher body mass index (BMI) could change make pressure sensor breathing signals less apparent. In contrast, while infrared thermography can accurately estimate breathing rates under challenging conditions such as motion and possible respiratory disorders, it is relatively expensive and also cannot provide information when obstacles cover the subject’s face.

Recently, IR-UWB radar has been used to continuously assess patient respiration and heart rates in a non-contact manner and to detect objects without interference from other sensors through the use of ultra-wideband frequencies. UWB systems are based on radio waves occupying a frequency band of >500 MHz or 25% of the fractional bandwidth. After the legalization of UWB by the FCC in 2002, UWB technology has been of great interest in various fields such as wireless communication and radar sensor applications^21–24^. With high carrier frequencies of at least 6 GHz, IR-UWB radar has a high spatial resolution for observation in indoor environments. IR-UWB radar is also harmless to the human body because it transmits and receives signals with very low power. Thus, it can be used for the detection of fine motions such as breathing or heartbeat^15,17^. A recent study showed that the IR-UWB radar sensor is precise and accurate for assessing heart rate and rhythm in a non-contact fashion^14^. Due to these characteristics, IR-UWB radar sensors show promise for the assessment of breathing patterns during sleep. A pilot study reported the accuracy of IR-UWB radar in terms of practical objective sleep assessment (architecture)^16^. However, this study enrolled a small sample (n = 12) and did not include control subjects. To our knowledge, the present study is the first to demonstrate the usefulness of IR-UWB radar for the detection of OSA. Moreover, our study had a sufficient sample size to evaluate the data according to OSA severity.

However, our study has some limitations. First, we collected IR-UWB radar data with a fixed distance between the radar and subject. Thus, additional validation is required in various setting environments. Second, this study did not consider information on sleep stages. If IR-UWB radar can diagnose sleep status, it will be more powerful for use in sleep medicine. Third, the IR-UWB radar sensor tended to be less specific when scoring respiratory effort-related arousals compared to scoring hypopnea and apnea. For this reason, we could not compare the agreement in respiratory disturbance index between PSG and IR-UWB radar.

In conclusion, the results of the present study demonstrated the feasibility of IR-UWB radar for the diagnosis of sleep apnea. Non-contact respiratory monitoring using IR-UWB radar accurately and reliably measured respiratory events during sleep. Therefore, IR-UWB radar may be a useful respiratory monitoring method that can overcome the difficulties of conventional PSG requiring physical contact with the patient. Further studies are needed to determine the data reproducibility of IR-UWB radar and its cost-effectiveness as a potential screening tool for sleep apnea.

## Supplementary information

Supplementary information. 
