# Supplementary material for: Non-contact diagnosis of obstructive sleep apnea using impulse-radio ultra-wideband radar
Source: Sci Rep. 2020 Mar 24;10:5261. doi: 10.1038/s41598-020-62061-4 (PMC7093464; doi:10.1038/s41598-020-62061-4)
Supplement: Supplementary file 1 — Supplementary information. [file 41598_2020_62061_MOESM1_ESM.docx]

**Non-contact diagnosis of obstructive sleep apnea using impulse-radio ultra-wideband radar**

**Sun Kang**^1+^**,** **Dong-Kyu Kim**^2+^**,** **Yonggu Lee**^3^**,** **Young-Hyo Lim**^3^**,** **Hyun-Kyung Park**^4^**,** **Sung Ho Cho**^1^***,** **and Seok Hyun Cho**^5^*

^1^Department of Electronics and Computer Engineering, Hanyang University, Seoul, Republic of Korea

^2^Department of Otorhinolaryngology-Head and Neck Surgery and Institute of New Frontier Research, Chuncheon Sacred Heart Hospital, Hallym University College of Medicine, Chuncheon, Republic of Korea

^3^Division of Cardiology, Department of Internal medicine, College of Medicine, Hanyang University, Seoul, Republic of Korea

^4^Department of Pediatrics, College of Medicine, Hanyang University, Seoul, Republic of Korea

^5^Department of Otorhinolaryngology-Head and Neck Surgery, College of Medicine, Hanyang University, Seoul, Republic of Korea

**^+^These authors contributed equally to this work**

***Corresponding authors:**

**Sung Ho Cho, PhD.**

Department of Electronics and Computer Engineering, Hanyang University, 222 Wangsimni-ro, Seongdong-gu, Seoul 04763, Republic of Korea

Telephone: +82-10-5412-5178, Fax: +82-2-2220-4883, Email: [dragon@hanyang.ac.kr](mailto:dragon@hanyang.ac.kr);

**Seok Hyun Cho, MD, PhD.**

Department of Otorhinolaryngology–Head and Neck Surgery, College of Medicine, Hanyang University, 222 Wangsimni-ro, Seongdong-gu, Seoul 04763, Republic of Korea

Telephone: +82-2-2290-8583, Fax: +82-2-2293-3335, Email: [shcho@hanyang.ac.kr](mailto:shcho@hanyang.ac.kr)

**Supplementary Table 1.** Agreement between Abnormal Breathing Index and Apnea–Hypopnea Index according to OSA severity.

|  | Mean ± SD | Limit of Agreement | |
| --- | --- | --- | --- |
|  |  | Upper | Lower |
| Normal (n=23) | -2.29±1.68 | 0.99 | -5.58 |
| Mild OSA (n=24) | -2.03±3.58 | 4.99 | -9.05 |
| Moderate OSA (n=14) | -2.27±4.89 | 11.87 | -7.33 |
| Severe OSA (n=33) | 9.46±12.24 | 33.46 | -14.53 |

OSA, Obstructive sleep apnea.
